# Brain Shift Patterns: Upward, Lateral and Downward Herniation, Its Correlation with Clinical Patterns in Acute Intracranial Pathologies and Neurosurgical Management

**DOI:** 10.21315/mjms-11-2025-787

**Published:** 2025-12-31

**Authors:** Nur Nazleen Said Mogutham, Jafri Malin Abdullah, Suzuanhafizan Omar, Mohammad Iskandar Sa’uadi, Sharifah Nawal Syed Jaafar

**Affiliations:** 1 Department of Neurosciences, School of Medical Sciences, Universiti Sains Malaysia, Health Campus, Kelantan, Malaysia; 2 Department of Neurosurgery, Tunku Abdul Rahman Neuroscience Institute, Hospital Kuala Lumpur, Kuala Lumpur, Malaysia; 3 Brain Behaviour Cluster, Universiti Sains Malaysia Specialist Hospital and School of Medical Sciences, Universiti Sains Malaysia, Health Campus, Kelantan, Malaysia; 4 Department of Neurosurgery, Hospital Sultanah Nur Zahirah, Kuala Terengganu, Terengganu, Malaysia; 5 Department of Neurosurgery, Hospital Sultanah Aminah, Johor Bharu, Malaysia

**Keywords:** brain shift, midline shift, transtentorial herniation, Glasgow Coma Scale, pupillary reactivity, intracranial pressure, neurosurgery

## Abstract

Brain shift is a critical radiological indicator of intracranial mass effect, resulting from haematoma, tumour, infarction, or cerebral oedema. Depending on the direction of displacement, herniation may occur as downward transtentorial, upward (reverse transtentorial), or lateral midline shift (MLS). This study aims to classify brain shift patterns and correlate radiological findings with clinical parameters, including level of consciousness, Glasgow Coma Scale (GCS), pupillary size and reactivity, and predicted intracranial pressure (ICP). Radiological data from CT and MRI were analysed alongside clinical observations in patients with acute intracranial pathology. Downward transtentorial herniation was graded into four stages based on caudal migration of the mammillary bodies, while upward transtentorial herniation was classified into three stages reflecting progressive cerebellar and brainstem displacement. Increasing degrees of brain shift correlated with declining GCS, pupillary abnormalities, and elevated ICP. In conclusion, standardised integration of imaging-based brain shift classification with clinical assessment enables earlier recognition, optimised surgical intervention, and improved prognostication in patients with acute intracranial pathologies.

## Introduction

Brain shift represents a key radiological indicator of intracranial mass effect and remains a critical determinant of neurological deterioration and prognosis in neurosurgical patients. It refers to the displacement of brain structures secondary to an increase in intracranial volume, commonly resulting from haematoma, tumour, infarction, or cerebral oedema. This displacement can manifest as midline shift (MLS), downward transtentorial (uncal) herniation, or upward transtentorial (central or ascending) herniation, depending on the direction and magnitude of pressure gradients within the cranial compartments.

MLS is typically measured as the horizontal displacement of midline structures, such as the septum pellucidum, which serves as a quantifiable radiological surrogate of raised intracranial pressure (ICP) and cerebral asymmetry. Multiple studies have demonstrated an inverse correlation between the extent of MLS and the Glasgow Coma Scale (GCS), highlighting its prognostic value in traumatic and non-traumatic intracranial lesions (1, 2). A shift exceeding 5 mm is often associated with significant neurological compromise and the risk of herniation syndromes (3).

Downward transtentorial herniation (DTH), also referred to as uncal herniation, occurs when medial temporal lobe structures are displaced inferiorly through the tentorial notch, compressing the midbrain and the ipsilateral third cranial nerve. This leads to progressive decline in consciousness, contralateral hemiparesis, and ipsilateral pupillary dilation due to oculomotor nerve compression (4).

Conversely, upward transtentorial herniation (UTH), a less frequent but equally fatal event, arises when posterior fossa lesions exert upward pressure through the tentorial hiatus, resulting in compression of the midbrain and diencephalic structures (5). Both syndromes reflect critical ICP gradients and share a common pathophysiological mechanism of brainstem distortion and impaired arousal pathways.

The clinical manifestations of brain shift are therefore intimately related to its radiological features. As displacement increases, the level of consciousness deteriorates due to reticular activating system compression, pupillary size and reactivity become altered from cranial nerve III involvement, and motor responses decline in tandem with descending corticospinal tract distortion (6). Despite these well-established pathophysiological relationships, limited studies have comprehensively analysed the correlation between different forms of brain shift: midline, upward, downward, and their combined impact on neurological status.

Understanding these correlations is vital for timely diagnosis, monitoring, and surgical decision-making. Integrating radiological indices of brain shift with clinical parameters, such as GCS and pupillary findings, can improve prognostic assessment and guide early intervention in patients with acute intracranial pathology. Hence, this study attempts to establish a classification system for radiological brain shifts secondary to acute intracranial pathologies and their associated clinical signs. In order to better understand the concept of herniation and its various types, a brief discussion on upward, horizontal, and downward herniation is provided.

## Downward Herniation

DTH, also known as central or descending tentorial herniation, occurs when increased ICP forces diencephalic and brainstem structures to shift caudally through the tentorial notch. This displacement leads to the compression of vital neural pathways, vascular compromise, and progressive neurological deterioration. Early recognition of this condition is crucial, as it represents a continuum from reversible diencephalic dysfunction to irreversible brainstem failure and death.

Radiological evaluation plays a pivotal role in diagnosing and staging DTH. Magnetic resonance imaging (MRI) and computed tomography (CT) reveal characteristic anatomic shifts that closely correspond to clinical findings. While CT remains the primary imaging modality in acute settings, MRI offers superior soft-tissue contrast and anatomical delineation of the mammillary bodies (MB), optic chiasm (OC), and tuber cinereum. The position of key neuroanatomical landmarks — such as the MB, OC, tuber cinereum, and splenium of the corpus callosum (CC) — serves as objective indicator of herniation severity. Progressive caudal migration of these structures, particularly relative to reference planes like the tuberculum venous confluence and incisural lines, marks the transition between stages.

Clinically, the evolution of DTH manifests through a predictable sequence of neurological and physiological changes. Early stages involve mild alteration in consciousness with preserved motor responses, while advanced stages are associated with decorticate and decerebrate posturing, fixed pupils, and respiratory irregularities. Ultimately, lower pontine and medullary involvement results in coma, flaccidity, and respiratory arrest.

This work synthesised radiological and clinical correlations across the four defined stages of DTH, highlighting imaging criteria and physiological responses that aid in clinical assessment and management. The staging framework presented here was drawn from prior radiological studies and clinical observations, notably those of Young et al. (7), Posner et al. (8), and Reich et al. (9), providing a comprehensive overview of the structural and functional progression of this critical neurological emergency.

## Stages of DTH

### Grade 1 (Minimal)/Early Diencephalic Stage

**Figure 1:**
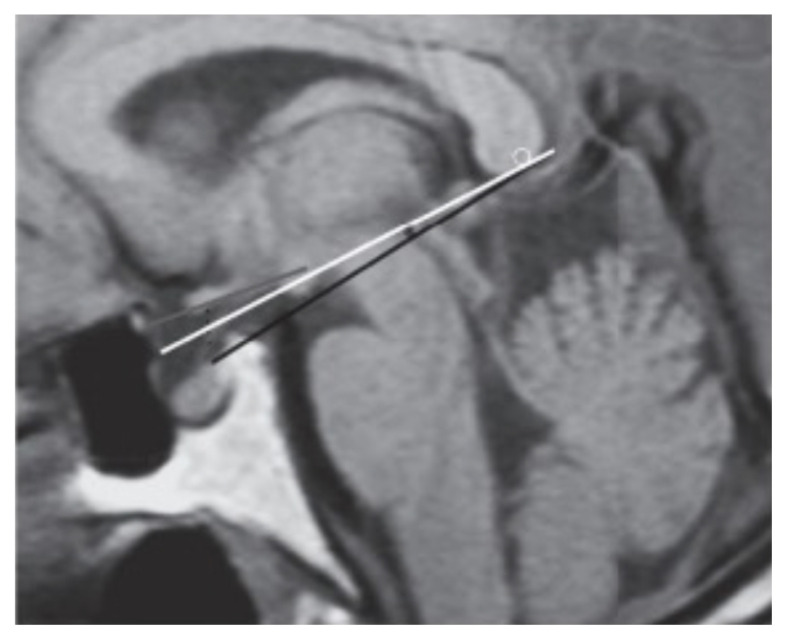
Grade 1 or early (minimal) central incisural herniation

In the earliest stage of DTH, a caudal displacement of the MB from their normal position occurs above the tuberculum venous confluence line to a location between this line and the incisural line. Concurrently, the interval between the OC and the plane of the diaphragma sellae (DS) becomes abnormally shortened. The long axis of the OC and visual tracts, which normally form a positive upward angle relative to the tuberculum venous confluence line, rotates into a parallel or slightly negative orientation. The distance between the splenium of the CC and the incisural line becomes essentially zero. All these findings are summarised in Figure 1 (7). Clinically, the patient presents with normal or slightly decreased mental status and maintains appropriate motor responses. The pupils are small but reactive, and vital signs remain normal.

Findings include caudal shift of the MB from their normal position located above the tuberculum venous confluence line to a position interposed between the tuberculum venous confluence line and the incisural line.

Reference lines:

- White line: tuberculum venous confluence line
- Black line: incisural line
- Black triangle line: interval between the OC and the plane of the DS
- Grey line: long axis of the chiasm/visual tract
- White circle: splenium of the CC

### Grade 2 (Moderate)/Late Diencephalic Stage

**Figure 2:**
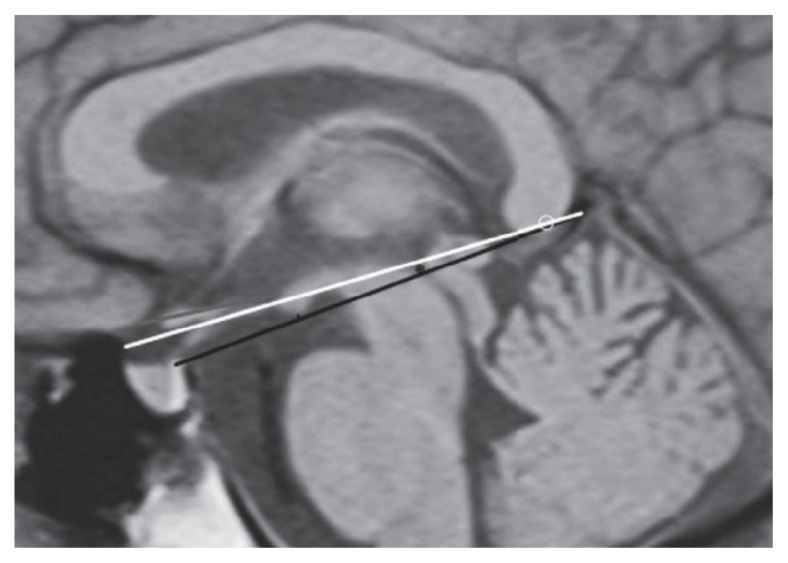
Grade 2 or moderate central incisural herniation

As herniation progresses, the MB shift further caudally to reach the incisural line. The interval between the MB and the belly of the pons diminishes, and the shape of this region transforms from quadrangular to triangular. The OC-DS interval continues to decrease, while the tuber cinereum changes from a straight to a sagging configuration. The long axis of the OC and visual tracts assumes a more negative angle. Both the iter and splenium, which were previously supratentorial, begin to approach the incisural line, as described in Figure 2 (7). Neurologically, mental status deteriorates, and patients exhibit decorticate posturing. Pupils remain small and reactive. Vital signs often demonstrate bradycardia, hypertension, and irregular or Cheyne-Stokes respiration.

Findings include progressive caudal shift of the MB from their grade 1 position just below the tuberculum venous confluence line to a position at the incisural line.

Reference lines:

- Grey line: long axis of the chiasm/visual tract
- White line: tuberculum venous confluence line
- Black snowflake: the iter position
- White circle: splenial positions
- Black line: incisural line

### Grade 3 (Advanced)/Mesencephalicupper Pontine Stage

**Figure 3:**
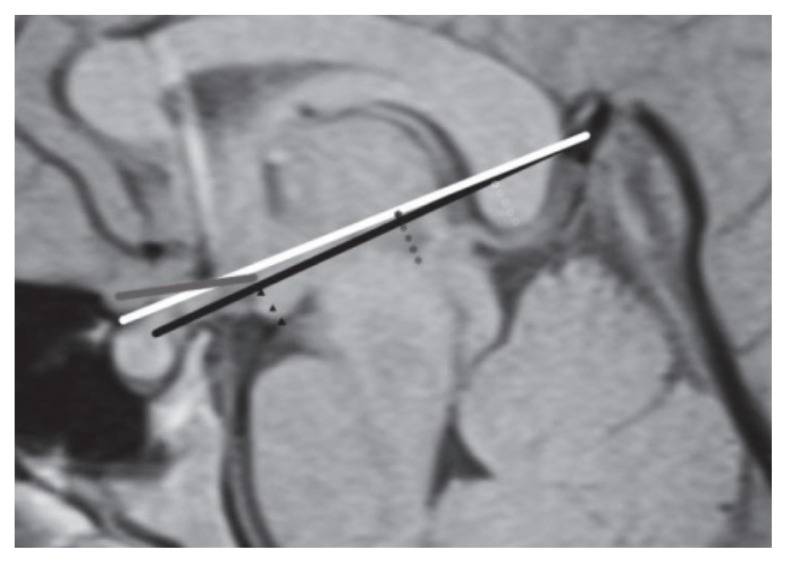
Grade 3 advanced central incisural herniation

In the advanced stage, the MB descend below the incisural line into an infratentorial position. The distance between the MB and the belly of the pons becomes markedly reduced, and the interpeduncular fossa takes on a distinctly triangular appearance. The OC nearly reaches the entrance of the sella, and the chiasm-visual tract axis becomes sharply negatively angled relative to the tuberculum venous confluence line. Both the iter and splenium have descended beyond the incisural line into the posterior fossa. The tuber cinereum sags noticeably and begins to buckle over the DS, an imaging hallmark of advanced central incisural herniation. The interpeduncular fossa becomes nearly effaced as the MB approach the belly of the pons (Figure 3) (7). Clinically, patients exhibit decreased consciousness with decerebrate posturing. The pupils are mid-position and fixed, and breathing patterns show Cheyne-Stokes respiration or apnoea.

Progressive caudal shift of the MB from their grade 2 position at or minimally below the incisural line to an infratentorial position.

Reference lines:

- Grey line: long axis of the chiasm/visual tract
- White line: tuberculum venous confluence line
- Black snowflake: the iter position
- White circle: splenial position
- Black line: incisural line

### Grade 4 (Very Advanced)/Lower Pontinemedullary Stage

**Figure 4:**
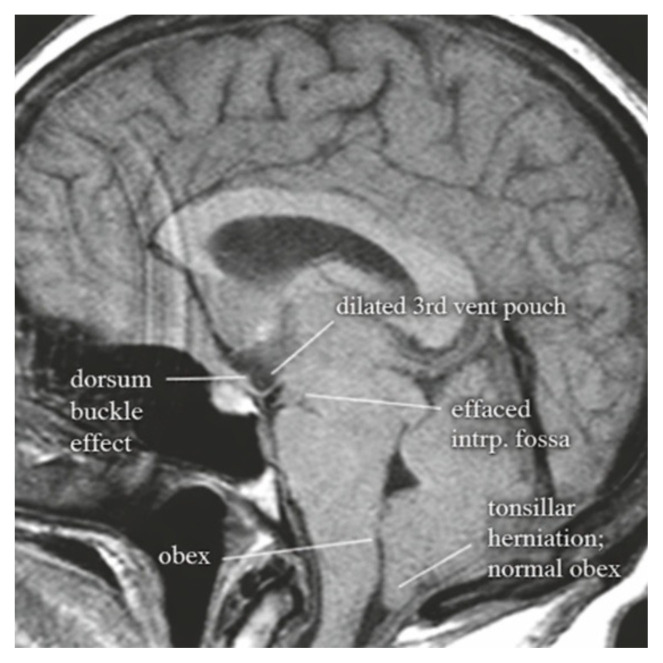
Very advanced grade 3 central incisural herniation

In the most advanced stage, the MB lie directly against the belly of the pons, completely effacing the interpeduncular fossa. The tuber cinereum markedly buckles over the DS, producing a cerebrospinal fluid pocket within the lower third ventricle. The suprasellar cistern is obliterated as the OC descends to the level of the sella. The chiasm-visual tract axis remains at a strongly negative angle relative to the tuberculum venous confluence line. Both the iter and splenium have moved well below the incisural line into the posterior fossa, while the obex lies low and the cerebellar tonsils become herniated beyond their normal limits (Figure 4) (7). Clinically, the patient presents with profound coma, flaccid motor responses, and fixed, dilated pupils. Respiratory patterns are characterised by Cheyne-Stokes respirations or apnoea.

In conclusion, DTH is a progressive and fatal intracranial event if not identified and managed promptly. The sequential imaging and clinical findings described across the four grades — ranging from early diencephalic displacement to pontomedullary compression — reflect the anatomical and physiological continuum of this process. Radiological recognition of subtle anatomic shifts, particularly the caudal migration of the MB and effacement of the interpeduncular cistern, plays a crucial role in early diagnosis.

Integration of clinical findings with imaging markers enhances diagnostic precision and enables early detection of subtle herniation, especially in cases of intracranial hypotension or diffuse cerebral oedema. Establishing a standardised radiologic staging system for DTH enhances diagnostic accuracy, facilitates communication among multidisciplinary teams, and contributes to better prognostic assessment. The progression through defined stages also offers a valuable educational tool for radiologists, neurologists, and critical care specialists in recognising and interpreting evolving herniation patterns (Table 1).

## Horizontal Herniation

Brain MLS can occur due to significant acute intracranial pathology, whether traumatic or non-traumatic. MLS is a “critical measurement for assessing brain symmetry changes and a vital indicator of pathological severity” (10). Early debate on the correlation between brain shift and consciousness level by Ropper (11) discussed the relevance of horizontal brain displacement versus transtentorial herniation in 24 patients with unilateral cerebral mass. He emphasised that “horizontal displacement at the level of pineal body was the earliest and most consistent distortion that affected patient’s reticular activating system” and that if the shift and level of consciousness were appropriate, surgery would be beneficial. In 1988, Ross et al. (12) further investigated this theory by adding a lateral shift of the aqueduct and septum. The study found that a decrease in the level of consciousness was associated with a significant increase in the mean lateral brain displacement at the pineal gland (from 3.8 to 7.0 mm) and septum pellucidum (from 5.4 to 12.2 mm). Nevertheless, they concluded that the pineal shift did not have strong predictive value for the restoration of consciousness level after surgery, and poor prognostic factors were a large septal shift and effacement of bilateral perimesencephalic cisterns.

Over the years, there has been ongoing research and development regarding this topic. Jacobs et al. (13) highlighted that unfavourable outcome after traumatic brain injury (TBI) was associated with “intracranial mass lesion, compression of perimesencephalic cisterns, MLS and traumatic subarachnoid haemorrhage (SAH)” (13).

Several attempts have been made to investigate the correlations among MLS and GCS, pupillary size, and ICP, with the intention of predicting patient prognosis. The earliest and most reliable finding was reported by Ropper (11), who revealed that “early depression of the level of alertness corresponded to distortion of the brain by horizontal displacement (pineal body) rather than transtentorial herniation with brainstem compression.” This simple correlation between MS measurement and the level of consciousness is highlighted in Table 2 based on the theory by Ropper (11, 14). Over the years, this theory has attracted other parameters worth investigating.

Meanwhile, Kim (15) discussed independent predictors of mortality and functional recovery in patients with intracerebral haemorrhage. He had broken down the GCS score into consciousness level and responsiveness, which paved the way for this study’s version of correlation between MLS and GCS scores (Table 3). Furthermore, bilateral non-reactive pupils were an independent predictor of 30-day mortality, but not a predictor of 90-day recovery. In 2013, Haque et al. (16) conducted a study on 64 patients. They reported a strong negative association between septum pellucidum shift and GCS score, as demonstrated on the scatter diagram. However, they used a broad range of shifts (less or more than 10 mm) and no specific GCS score.

With regards to the impact of MLS, pupillary size, and reactivity plus ICP, Taylor et al. (17) reported that the resting pupillary size averaged at 4.1 mm and the constriction velocity (CV) averaged at 1.48 ± 0.33 mm/second. Eight patients with raised ICP above 20 mm Hg had normal pupillary dynamics. Once the CV fell below 0.6 mm/second, it is likely that ICP, “if not already elevated, will become elevated, within 15 to 30 minutes in patients with a significant mass effect.” This explains the choice of more than 4 mm for anisocoria in this study, as demonstrated in Table 4. McNett et al. (18) argued that “spikes in ICP values resulted in corresponding variations in paired pupillometer values for pupillary CV and Neurological Pupil Index (NPi), but not necessarily for pupil size” (19). This notion was supported by Osman et al. (20), who conducted a study on 134 patients with ischaemic and haemorrhagic stroke (21).

The estimation of ICP value and its association with MLS was discussed by Chen et al. (22), whereby a prediction model was designed using RapidMiner to estimate ideal MLS and ICP levels to be used as efficient prescreening for ICP monitoring. However, they used only two measurement groups: ICP > 12 or ≤ 12, which is not specific. This study referred to Taylor et al. (17) to predict the model presented in Table 3. According to Taylor et al. (17), “CV decreased to below 0.8 mm/second when the ICP exceeded 30 mm Hg in 8 of 34 paired observations. CV fell below 0.6 mm/second, and the percentage of reduction in pupil size fell below 10% only when the ICP exceeded 35 mm Hg.”

A study on TBI by Palekar et al. (23) went a step further and looked at MLS and its correlation with GCS, pupillary reaction, and Glasgow Outcome Score. They found that 35.4% of their 48 patients with a higher degree of MLS had a poor outcome. It was also noted that patients with MLS > 5 mm had a higher percentage of unequal pupils and also bilaterally non-reactive pupils. This was associated with a statistically significant poor outcome (*P* = 0.008) (23) despite no specific size being mentioned.

In 2003 Englander et al. (24) put forth a theory that MLS of more than 5 mm on CT scans is associated with poorer outcomes in terms of assisted ambulation and “global supervision at rehabilitation discharge.” Chiewvit et al. (25) supported this negative correlation and added that the poorest outcome was those with MLS and subdural haemorrhage as the acute intracranial pathology. Interestingly, the correlation between the lower GCS (≤ 12) and the degree of MLS was not statistically significant (23).

## Upward Herniation

UTH was first described in 1948 by Ecker (26). Since then, multiple authors have highlighted its importance and proposed various terms, including reverse brain herniation (27), ascending transtentorial herniation (28), and upward cerebellar herniation (29). The term UTH was preferred in this study since it is self-descriptive. However, care must be taken not to confuse it with upward “transcalvarial” herniation, which refers to herniation of the brain through a surgical defect (30, 31).

In essence, UTH occurs when increased pressure within the posterior fossa forces cerebellar and brainstem structures upward through the tentorial incisura, resulting in compression of vital midbrain structures (29). Unlike the more commonly discussed downward herniation syndromes, UTH presents unique challenges in diagnosis and management due to its rapid progression and potentially devastating consequences.

The pathophysiology involves both direct mechanical compression and secondary vascular compromise of penetrating arteries supplying the midbrain and upper brainstem. This dual mechanism leads to progressive neurological deterioration that can be categorised into distinct stages, each with characteristic imaging findings, clinical symptoms, and therapeutic implications.

To date, other than the clinical description of UTH (9, 26–30), no previous study has described in detail the stages of UTH. Therefore, this study aims to illustrate the stages in a table outlining their characteristic imaging findings and associated clinical symptoms.

### Pathophysiology and Anatomical Considerations

The tentorial incisura serves as a natural anatomical boundary between the supratentorial and infratentorial compartments. When posterior fossa pressure increases due to mass lesions, haemorrhage, or oedema, the superior cerebellar structures are forced upward through this opening (6). The key anatomical structures involved include:

- Superior cerebellar vermis: The first structure to be displaced upward
- Midbrain: Compressed against the tentorial edge
- Quadrigeminal cistern: Early site of compression and obliteration
- Posterior third ventricle: May become compressed in advanced stages
- Cerebral aqueduct: Can become obstructed, leading to hydrocephalus

The vascular supply to these regions, particularly the superior cerebellar arteries and perforating branches to the midbrain, becomes compromised during the herniation process, leading to ischaemic changes that may progress to haemorrhagic transformation (Duret haemorrhages) in severe cases (32).

### Progressive Stages of UTH

UTH is classified into three distinct clinical stages: mild, moderate, and severe, each reflecting progressive compression of neural structures, worsening neurological dysfunction, and corresponding neuroimaging findings. The stages are summarised in Table 4.

#### Early Stage (Mild Compression)

The early stage of UTH is characterised by initial compression of the superior cerebellar vermis and mild distortion of the quadrigeminal cistern. This stage represents the earliest detectable changes that may precede obvious clinical deterioration.

##### Displacement of anatomical structures

The primary structures affected during this stage include the superior cerebellar vermis and the midbrain region. The vermis begins to show upward displacement, while the midbrain experiences initial contact with the tentorial edge (33).

##### Neuroimaging findings

CT and MRI reveal characteristic early changes. The most significant finding is the flattening of the quadrigeminal cistern, which appears compressed on axial imaging (26). SAH may be present in the cerebellar folia, creating a “zebra sign” pattern with associated vasogenic oedema and effacement of the fourth ventricle (34). Early signs of hydrocephalus may be evident, and importantly, the iter (cerebral aqueduct) measures less than 3mm above the incisural line, indicating an initial upward displacement (9).

##### Clinical manifestations

Patients in the early stage typically remain alert but may experience subtle neurological changes. Common symptoms include nausea, vomiting, and occipital headache, which reflect increased ICP. Diplopia, vertigo, and ataxia may develop as cerebellar function becomes compromised. Subtle neurological changes become apparent, including altered eye movements and slight drowsiness. These early signs are often nonspecific but should raise suspicion in patients with known posterior fossa pathology.

##### Pupillary and vital sign changes

During the early stage, pupils typically remain symmetrical and reactive to light, indicating preserved brainstem function. Vital signs generally remain normal, as significant brainstem compression has not yet occurred.

#### Mid-stage (Increasing Compression)

The mid-stage represents significant progression with involvement of multiple brainstem structures and more obvious clinical deterioration. This stage requires urgent intervention to prevent further deterioration.

##### Anatomical structures displaced

The compression extends to involve the cerebellum more extensively, along with the midbrain, pineal gland, pons, and cerebrospinal fluid (CSF) flow pathways. The degree of structural displacement becomes more pronounced and clinically significant (35).

##### Neuroimaging findings

Imaging studies reveal more extensive changes during this stage. Cerebellar oedema becomes more prominent, and there is notable angulation or kinking of the quadrigeminal plate (35). The aqueduct of Sylvius shows signs of obliteration, contributing to the development of marked hydrocephalus. The flattening of the ventral pons becomes apparent, and posterior flattening of the third ventricle may be observed. The pineal gland appears distorted and measures less than 1cm in diameter. Critically, the iter now measures 3 to 7 mm above the incisural line, indicating significant upward displacement (9).

##### Clinical manifestations

Neurological deterioration becomes more apparent during this stage. Patients experience a decreased level of consciousness, progressing from lethargy to stupor. Oculomotor nerve dysfunction becomes evident, and there is a possible development of Parinaud syndrome, characterised by upward gaze palsy and other dorsal midbrain signs. Brainstem reflexes become incomplete, indicating significant brainstem compromise. Decerebrate posturing may develop due to upper midbrain damage, and hyperreflexia with bilateral Babinski responses may be present.

##### Pupillary and vital sign changes

The pupils become medium-sized (4 to 6 mm) and may show reduced reactivity to light. Vital signs begin to show abnormalities, including bradycardia, hypertension, and irregular breathing patterns consistent with Cushing’s triad. Cheyne-Stokes respiration may develop, indicating involvement of the respiratory centres.

#### Late Stage (Severe Compression)

The late stage represents the most critical phase, with severe brainstem compression and potential for imminent brainstem herniation and death. This stage requires immediate life-saving intervention.

##### Anatomical structures displaced

Extensive involvement includes the cerebellum, midbrain, pons, pineal gland, vein of Galen, basal vein of Rosenthal, and CSF flow pathways. The degree of displacement becomes life-threatening.

##### Neuroimaging findings

Imaging reveals severe pathological changes. Cerebellar oedema is extensive, along with complete obliteration of the aqueduct of Sylvius. Gross hydrocephalus develops as CSF flow becomes severely compromised. The flattening of the ventral pons becomes pronounced, and the pineal gland shows severe distortion, measuring greater than 1cm. Most critically, the iter measures greater than 7 mm above the incisural line, indicating severe upward displacement that threatens brainstem viability (9).

##### Clinical manifestations

Patients progress to deep coma with decerebrate posturing due to upper pons damage. Abnormal respiratory patterns develop, typically Cheyne-Stokes respiration or apnoea, indicating severe compromise of respiratory centres. Brainstem reflexes become absent, suggesting imminent brainstem death (8).

##### Pupillary and vital sign changes

Pupils become fixed and dilated, indicating complete loss of brainstem function. Vital signs show Cheyne-Stokes respiration or apnoea, representing failure of central respiratory drive. Without immediate intervention, this stage progresses rapidly to brain death.

## Neurosurgical Management

In supratentorial brain injury, decompressive hemicraniectomy allows for rapid outward (transcalvarial) brain expansion, reducing the midline brain herniation and limiting secondary brain injury. The indicators to predict an optimum decompression are preoperative MLSs, craniectomy size, extent of transcalvarial herniation, and thickness of the tissues overlying the craniectomy (36).

Different techniques of decompressive craniectomy (DC) described in the literature, apart from the conventional technique, include hinge craniotomy (HC), decompressive craniectomy combined with temporal lobectomy, and controlled decompression techniques (37–39). A systematic review by Panchal et al. (37) revealed that HC can achieve decompression comparable to DC in both TBI and stroke patients. This is revealed by means of postoperative ICP, and intracranial volume expansion did not significantly differ between HC and DC (37).

A better outcome was also reported with the controlled decompression technique, with lower postoperative complications, reduced postoperative brain swelling, delayed haematoma formation, improved neurological outcome, and lower mortality (38).

In addition to that, decompressive craniectomy with temporal lobe resection may provide better decompression, more stable ICP control, lower mortality, and improved functional outcomes compared with standard DC in patients with massive cerebral infarction, as reported by Lu et al. (39). The detailed technique of cerebellar hemispherectomy in combating transtentorial herniation is further discussed.

## Cerebellar Hemispherectomy

### Position and Incision

**Figure 5:**
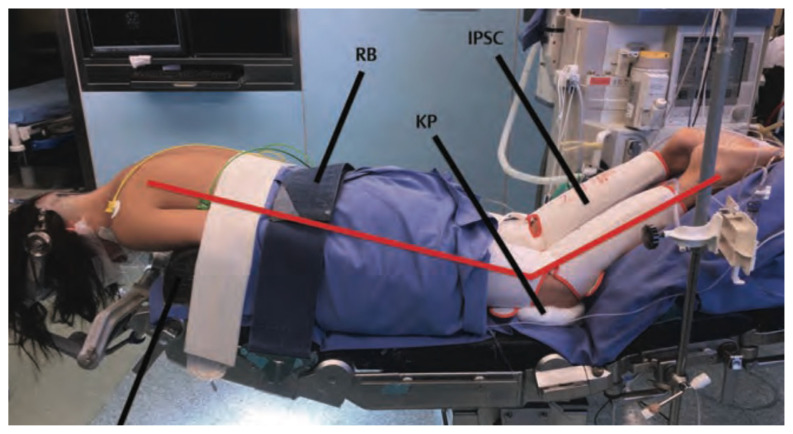
Patient in prone position and reverse Trendelenburg RB = restraint belt; KP = knee padding; IPSC = intermittent pneumatic compression Image source: Gagliardi et al. (40)

Patient was in the prone position with the head fixed to a Mayfield 3-pin clamp. The head was maintained straight and flexed, leaving a space of about 2 cm between the chin and the chest. The patient’s body was placed in a slight “Concorde” position with 10° to 20° of reverse Trendelenburg. The inion was at the highest point in the surgical field (Figure 5) (40).

**Figure 6:**
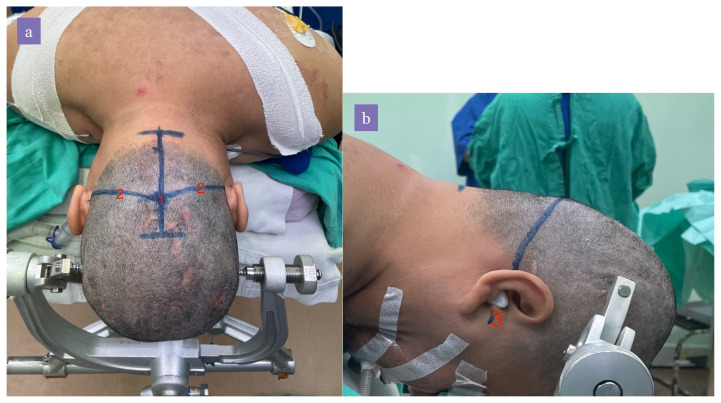
Craniometric marking and line 1 = inion; 2 = zygoma-inion line; 3 = zygomatic root

The inion and craniometric line of the zygoma-inion line, which is commonly used as the surface landmark for the transverse sinus, were marked (Figure 6).

**Figure 7:**
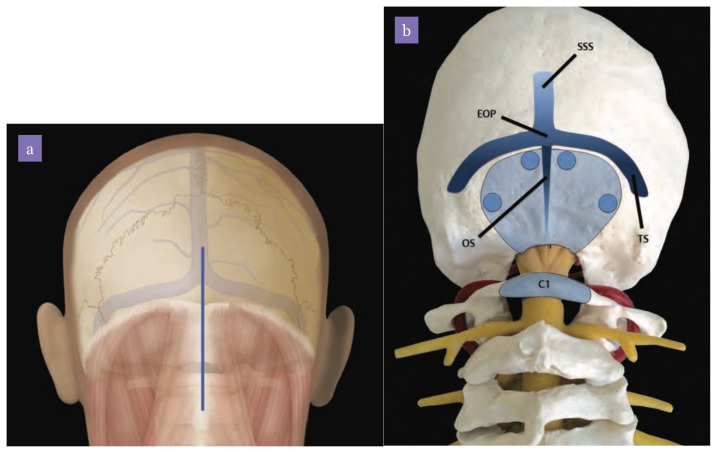
a) Illustrative image of skin incision and underneath structures; b) Illustrative image of burrholes and suboccipital craniotomy with C1 laminectomy Image source: Gagliardi et al. (40)

A straight midline skin incision with the starting point at 3 cm above the inion was made; the line runs exactly along the midline and ends at the spinous process of the axis (C2) (Figure 7).

### Craniotomy and Durotomy

Soft tissue was dissected at the midline at an avascular plane following skin incision. Origins of both trapezius, semispinalis capitis, rectus capitis posterior minor, and rectus capitis posterior major muscles were detached from the occipital bone and reflected laterally.

Subperiosteal dissection was performed of the occipital squama laterally from the midline to both sides to expose the occipital bone until the level of the C1 vertebrae posterior arch.

Four burr holes were made, followed by suboccipital craniotomy with or without C1 laminectomy. The usual borders of craniotomy are as follows: i) Laterally: craniotomy extends about 3.5 cm paramedian at the level of the inferior nuchal line; ii) Superiorly: 1 cm below the superior nuchal line, and iii) Inferiorly: Inferior margin of sub-occipital craniotomy is the magnum foramen (Figure 7) (40).

Dura was opened in a Y-shaped fashion, and the ligation of the occipital sinus and dura flaps is reflected laterally and superiorly to expose the bilateral cerebellar hemispheres.

### Hemispherectomy

**Figure 8:**
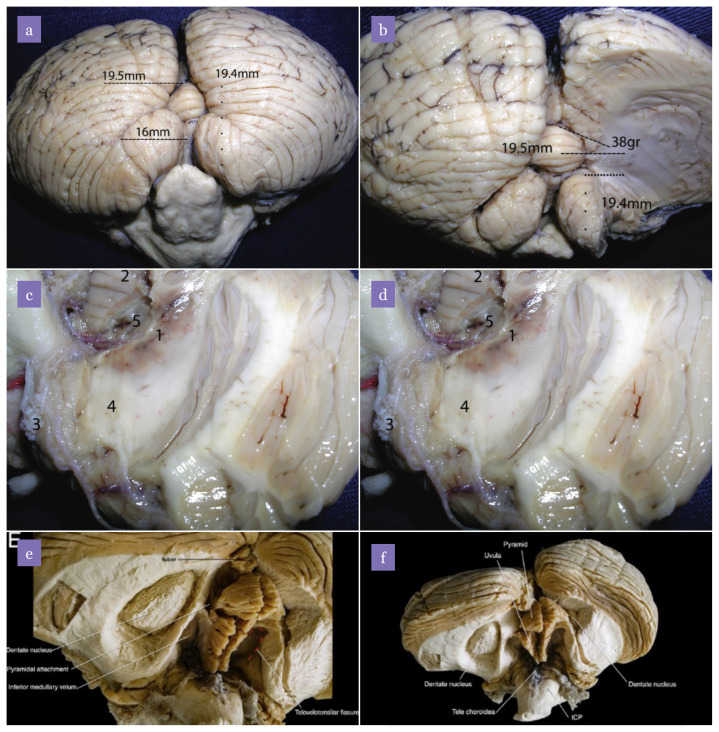
a–e) View from suboccipital surface; f) Bilateral biventral lobules hemisphere resected with the pyramid and uvula were sectioned at the midline Image source: Ramos et al. (41) and García-Feijoo et al. (42)

From the suboccipital surface (Figure 8) (41, 42):

- Dissection starts at the cerebellomedullary fissure and extends to the telovelotonsilar fissure. The superior limit of resection is 2 cm from the base (at the televelotonsilar cleft, as the dentate nucleus is located on average at 19.4 mm superior to the inferior border of the tonsil) (Figure 8a).
- Resection was continued rostrally with a medial border of 2 cm from the midline vermis (as the lateral border of the dentate nucleus is about 19.5 mm from the midline). Figure 8b shows the different relationships of the dentate nucleus to the nearby structures. 38º is, on average, the angle of separation from the midline to lateral border of the dentate nucleus;
- Left cerebellar hemisphere and tonsil are removed to expose the uvula and their relationship with the superior pole of the tonsil and the dentate nucleus. 1: dentate nucleus; 2: uvula; 3: flocculus; 4: middle cerebellar peduncle; 5: superior pole place of the tonsil after removed (Figure 8c).
- Complete resection of the right biventral lobule is performed. The tonsillar peduncle was preserved and the inferior fibres of the middle cerebellar peduncle that surround the DN were dissected (Figure 8d). Detailed view of the DN that shows its medial relation with the telovelotonsillar fissure (Figure 8e).

**Figure 9:**
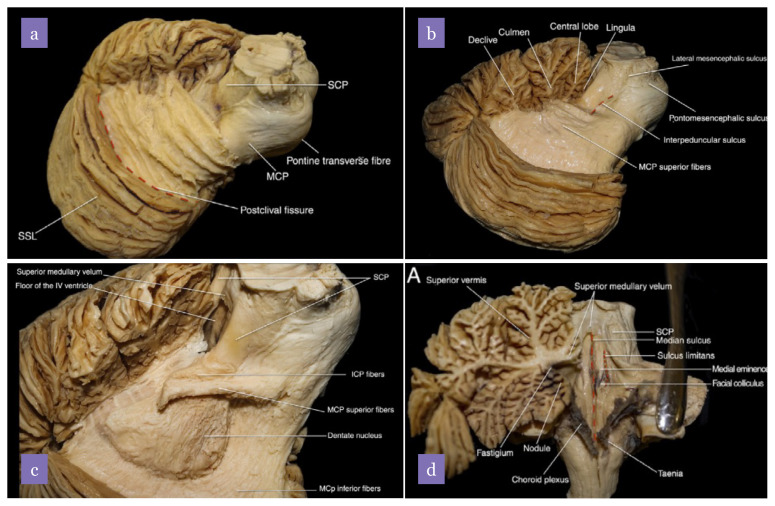
Tentorial surface: a–c) Lateral view of cerebellum with wrinkled appearance after the initial resection of the cerebellar lobes, with sulci corresponding to the disposition of the terminal white fibres in folia. White fibre dissection progress: the superior fibres of the inferior cerebellar peduncle cover the DN and travel posteriorly and medially. Detailed view of the DN, preserving a fragment of superior fibres of the MCP and a small remnant of inferior cerebellar peduncle fibres, emerging proximal to the hilum; d) Posterior view of the fourth ventricle, preserving the vermis, closely connected to the roof, in which a vertical section was performed. Dissector displacing the right DN Image source: García-Feijoo et al. (42)

From the tentorial surface (Figure 9) (42):

- Resection starts from the simple and quadrangular lobules, the dissection was lateral to the vermian-hemispheric line, with the cerebellomesencephalic fissure as the anterior border, followed by the cerebellopontine fissure. This will subsequently delineate the cerebellar peduncles.
- The juncture of the superior cerebellar peduncle (SCP), middle cerebellar peduncle (MCP), and inferior cerebellar peduncle, which form a posterolateral anatomical complex of the brainstem, was visualised and preserved. SCP was the easiest to identify on the tentorial face. Its medial edge borders the superior medullary velum, forming the superior half of the lateral wall of the fourth ventricle roof.
- The dentate nucleus was buried in the thickness of the MCP fibres, which was the thickest of the cerebellar peduncles. It adopted a triangular shape, with the vertex towards the hilum and the posteroinferior base.
- From the tentorial surface, the dentate nucleus was located about 18 mm from the highest part of the culmen. The medial limit was 1.5 cm from the midline vermis.
- SCP, MCP, inferior cerebellar peduncle, and vermis were the structures to be preserved. Hemispheric vein (superior, middle, and inferior) and distal branches of the inferior trunk of the superior cerebellar artery can be sacrificed during resection.

## Conclusion

Brain shift represents a dynamic and multifaceted manifestation of intracranial mass effect, encompassing horizontal, downward, and UTH, each with distinct radiological signatures and predictable clinical correlates. This review underscores the central role of neuroimaging in identifying subtle anatomical displacements that precede irreversible neurological deterioration. Across all herniation patterns, progressive distortion of critical neuroanatomical landmarks parallels declining levels of consciousness, pupillary abnormalities, and worsening motor responses, reflecting escalating compromise of the reticular activating system, cranial nerve pathways, and corticospinal tracts.

DTH demonstrates a well-defined anatomical and physiological continuum, in which staged caudal migration of the MB, OC, tuber cinereum, and CC reliably mirrors clinical progression from diencephalic dysfunction to pontomedullary failure. Establishing a structured radiological staging system for DTH enhances early recognition, facilitates interdisciplinary communication, and supports timely escalation of management before irreversible brainstem injury occurs.

Horizontal brain shift, most commonly quantified by septum pellucidum displacement, remains a robust surrogate marker of ICP gradients and supratentorial mass effect. The strong inverse correlation between MLS and GCS, along with its association with pupillary abnormalities, raised ICP, and poor functional outcomes, reinforces its prognostic value. However, the variability in outcomes highlights the need to interpret MLS in conjunction with cisternal status, pupillary dynamics, and clinical trajectory rather than as an isolated metric.

UTH, although less frequently described, represents an equally catastrophic process, particularly in posterior fossa pathology. This study contributes to the literature by delineating distinct radiological and clinical stages of UTH, using measurable anatomical landmarks such as the position of the iter relative to the incisural line. Recognition of early imaging changes—before overt brainstem failure—offers a critical window for intervention in a condition traditionally associated with high mortality.

From a therapeutic perspective, surgical decompression remains the cornerstone of management across herniation syndromes. While decompressive craniectomy and its variants are well established for supratentorial pathology, posterior fossa decompression and cerebellar hemispherectomy play a vital role in mitigating life-threatening upward and downward herniation. A precise understanding of herniation anatomy is essential to guide surgical strategy, optimise decompression, and minimise secondary injury to preserved brainstem structures.

In summary, integrating detailed radiological assessment with clinical examination allows for a unified framework to understand, classify, and manage brain shift syndromes. Standardised staging systems for horizontal, downward, and upward herniation not only improve diagnostic accuracy and prognostication but also support timely, anatomy-driven surgical decision-making. Future prospective studies correlating imaging-based staging with outcomes may further refine these frameworks and enhance care for patients with acute intracranial pathology.

## Figures and Tables

**Table 1:** Grades of DTH

Stages	Image findings and structure involved	Symptoms/neurological	Pupils/eye finding Vital signs
Grade 1 (minimal)/early diencephalic	Caudal shift of the MB from their normal position located above the tuberculum venous confluence line (white reference line) to a position interposed between the tuberculum venous confluence line (white reference line) and the incisural line (black reference line) Concurrently, the interval between the OC and the plane of the diaphragm sella (black triangle line) is shortened beyond the normal range The long axis of the chiasm/visual tract (grey line) rotates from a positive upward angle relative to the tuberculum venous confluence line (white line) into an orientation which is either parallel or at a slightly negative angle The splenium of the CC (white circle) to incisura line (black reference line) distance is essentially zero	Normal or decreased mental status with appropriate motor response	Small, reactive Normal
Grade 2 (moderate)/late dienephalic	Progressive caudal shift of the MB from their grade 1 position just below the tuberculum venous confluence line (white reference line) to a position at the incisural line (black reference line) The distance interval between the mammillary body and the belly of the pons is now noticeably diminished, and its shape is altered from a quadrangular to a triangular appearance The optic chiasm/diaphragm sella interval continues to decrease. The tuber cinereum changes from a straight line into a caudal sagging shape The long axis of the chiasm/visual tracts is now oriented at a negative angle (grey reference line) The iter (black snowflake) and splenial (white circle) positions, formerly supratentorial, now approach the incisural line (black reference line)	Decreased mental status with decorticate motor response	Small, reactive Bradycardia Hypertension Irregular breathing/Cheyne-Stokes
Grade 3 (advanced)/mesencephalic-upper pons	Progressive caudal shift of the MB from their grade 2 position to an infratentorial position (black triangle distance) The interval distance between the mammillary body and the belly of the pons is now significantly diminished, and the interpeduncular fossa is clearly triangular The OC has nearly reached the entrance of the sella. The long axis of the chiasm/visual tracts (grey reference line) is now at a significantly negative angle relative to the tuberculum venous confluence line (white reference line). The iter position and the splenium have crossed beyond the incisural line into the posterior fossa The tuber cinereum clearly sags inferiorly and begins to buckle over the dorsum sella (another characteristic sign of advanced central incisural herniation) The interpeduncular fossa is close to being completely effaced as the MB approach the belly of the pons	Decreased mental status with decerebrate motor response	Midpoint, fixed Cheyne-Stokes/apnoea
Grade 4 (very advanced)/lower pons-medullary	Notice that in this most advanced state of central incisural herniation, the MB have reached the belly of the pons completely effacing the interpeduncular fossa The tuber cinereum typically buckles over the dorsum sella, which creates a pocket of CSF within the lower third ventricle The suprasellar space is effaced as the OC position reaches the entrance to the sella The long axis of the chiasm/visual tracts is oriented at a significantly negative angle relative to the tuberculum venous confluence line The iter and the splenium have crossed the incisural line well into the posterior fossa The obex position is low, and the cerebellar tonsils are also herniated beyond the normal range	Decreased mental status with flaccid motor response	Fixed and dilated pupils Cheyne-Stokes/apnoea

OC = optic chiasm; CC = corpus callosum; CSF = cerebrospinal fluid; MB = mammillary bodies

**Table 2:** Effect of lateral shift on level of consciousness (14)

Amount of MLS (mm)	Level of consciousness
0 to 3	alert
3 to 4	drowsy
6 to 8.5	stuporous
8 to 13	comatose

MLS = midline shift

**Table 3:** Proposed relationship between MLS, level of consciousness, GCS, pupillary size and predicted ICP readings

MLS (mm)	Level of consciousness	GCS	Pupillary size (mm)	ICP (mmHg)
0 to 3	Alert	14 to 15	2 to 4	8 to 20
3 to 4	Drowsy	11 to 13	2 to 4	< 20
6 to 8.5	Stuporous	Obtunded: 9 to 10 Stuporous: 6 to 8	Anisocoria (> 4 on one side)	30 to 35
8 to 13	Comatose	3 to 5	Dilated bilaterally (> 4)	> 35

MLS = midline shift; GCS = Glasgow Coma Scale; ICP = intracranial pressure

**Table 4:** Grading of UTH

Stages	Structures displaced	Image findings	Symptoms/neurological	Pupil/eye findings Vital signs
Early Mild compression	Superior cerebellar vermis Midbrain	Flattening of the quadrigeminal cistern (6) Subarachnoid haemorrhage in the cerebellar folia (zebra sign) with vasogenic oedema and effacement of the fourth ventricle (post supratentorial CSF removal) Early sign of hydrocephalus Iter <3 mm above incisural line (7)	Alert Nausea/vomiting/occipital headache Diplopia/vertigo/ataxia Subtle neurological changes Altered eye movements Slight drowsiness	Symmetrical, reactive, nystagmus Normal
Mid Increasing compression	Cerebellum Midbrain Pineal gland Pons CSF flow	Cerebellar oedema Angulation or kinking of the quadrigeminal plate (6, 7) Obliteration of aqueduct of Sylvius Marked hydrocephalus Flattening of ventral pons Posterior flattening of 3rd ventricle Pineal gland distorted < 1 cm Iter 3 to 7 mm above incisural line (7)	Decreased level of consciousness/lethargy/stupor Oculomotor nerve dysfunction Possible development of Parinaud syndrome Brainstem reflex – incomplete Decorticate posturing – due to upper midbrain damage Hyperreflexia Bilateral Babinski upgoing	Medium (4 to 6 mm) pupils Bradycardia, hypertension, irregular breathing/Cheyne-Stokes
Late Severe compression (potential brainstem herniation and imminent death)	Cerebellum Midbrain Pons Pineal gland Vein of Galen Basal vein of Rosenthal CSF flow	Cerebellar oedema Obliteration of aqueduct of Sylvius Gross hydrocephalus Flattening of ventral pons Pineal gland distorted > 1 cm Iter > 5 mm above the incisural line with associated downward herniation (7)	Deep coma with decerebrate posturing – due to upper pons damage Abnormal respiratory patterns (Cheyne-Stokes or apnoea) Absent brainstem reflex	Fixed, dilated pupils Cheyne-Stokes/apnoea

CSF = cerebrospinal fluid
